# Supplementary material for: Mineral Composition of Skeletal Elements in Dorid Nudibranchia Onchidoris muricata (Gastropoda, Mollusca)
Source: Biomimetics (Basel). 2025 Mar 29;10(4):211. doi: 10.3390/biomimetics10040211 (PMC12025115; doi:10.3390/biomimetics10040211)
Supplement: Supplementary file 1 [file biomimetics-10-00211-s001.zip › Tables S1-S3.pdf]

Table S1. Supplementary. Dataset of the elemental composition of the *Onchidoris muricata* spicules in sections. The spicules belonging to clusters 1, 2, and 3 are colored green, blue, and peach, respectively.

Original name of a spicule	Specimen	Index number of a spicule	Cluster	Ca	Mg	O	C	Traces	P	Cl	F
**o27_1_sm**	o27	1	SC I	0.1457	0.0181	0.452	0.2642	0.1201	0.1061	0.004	0.01
**o27_2_sm**	o27	2	SC I	0.2396	0.0145	0.4498	0.1695	0.1265	0.1162	0.0023	0.008
**o27_3_sm**	o27	3	SC I	0.2138	0.0135	0.4623	0.1939	0.1164	0.102	0.0042	0.0102
**o27_4_sm**	o27	4	SC I	0.2042	0.0156	0.4477	0.2194	0.113	0.1014	0.0027	0.0089
**o27_5_sm**	o27	5	SC I	0.1942	0.0171	0.48	0.1786	0.1302	0.1158	0.0024	0.012
**o27_6_sm**	o27	6	SC I	0.1767	0.0173	0.4549	0.2387	0.1123	0.0986	0.0033	0.0104
**o27_7_sm**	o27	7	SC I	0.2378	0.0101	0.416	0.2036	0.1324	0.1186	0.0066	0.0072
**o27_8_sm**	o27	8	SC I	0.2301	0.0182	0.4339	0.1774	0.1404	0.1281	0.0038	0.0085
**o27_9_sm**	o27	9	SC I	0.2391	0.0139	0.4273	0.1764	0.1433	0.1311	0.0045	0.0077
**o27_10_sm**	o27	10	SC I	0.2449	0.0121	0.4331	0.1725	0.1374	0.1265	0.004	0.0069
**o27_11_sl**	o27	11	SC II	0.2131	0.0335	0.538	0.1809	0.0345	0	0.0012	0.0333
**o27_12_sl**	o27	12	SC III	0.2703	0.0329	0.537	0.1393	0.0206	0	0.0015	0.0191
**o27_13_sl**	o27	13	SC II	0.221	0.0518	0.5457	0.1388	0.0426	0	0.0017	0.0409
**o27_14_sl**	o27	14	SC II	0.1809	0.0504	0.6333	0.0954	0.0401	0	0.0012	0.0389
**o27_15_sl**	o27	15	SC II	0.2256	0.0403	0.5801	0.1101	0.0439	0	0.0016	0.0423
**o27_16_sl**	o27	16	SC II	0.1605	0.0498	0.6318	0.1049	0.0529	0	0.0011	0.0518
**o27_17_sl**	o27	17	SC II	0.1722	0.0309	0.6221	0.1538	0.021	0	0.0013	0.0197
**o27_18_sl**	o27	18	SC II	0.1972	0.0289	0.6113	0.141	0.0216	0	0.0012	0.0204
**o27_19_sl**	o27	19	SC II	0.2064	0.0515	0.6128	0.0881	0.0413	0	0.0014	0.0399
**o27_20_sl**	o27	20	SC II	0.2009	0.0299	0.6123	0.1263	0.0306	0	0.0014	0.0292
**o27_21_sm**	o27	21	SC I	0.1523	0.0188	0.4932	0.213	0.1227	0.1072	0.0027	0.0128
**o27_22_sm**	o27	22	SC I	0.1469	0.0206	0.5028	0.2098	0.1199	0.1059	0.0026	0.0114
**o27_23_sm**	o27	23	SC I	0.1593	0.0205	0.518	0.1985	0.1036	0.0875	0.0032	0.0129
**o27_24_sm**	o27	24	SC I	0.2164	0.0165	0.4447	0.1983	0.1242	0.1116	0.0034	0.0092
**o27_25_sm**	o27	25	SC II	0.266	0.0344	0.6092	0.0741	0.0163	0	0.0011	0.0152
**o27_26_bl**	o27	26	SC II	0.1998	0.0406	0.6468	0.0835	0.0293	0	0.0013	0.028
**o27_27_bl**	o27	27	SC II	0.1788	0.0295	0.6626	0.1113	0.0179	0	0.0011	0.0168
**o27_28_bl**	o27	28	SC II	0.1854	0.0353	0.6519	0.1072	0.0202	0	0.001	0.0192
**o27_29_bl**	o27	29	SC II	0.1862	0.0464	0.6466	0.0899	0.0308	0	0.001	0.0298
**o27_30_bl**	o27	30	SC II	0.195	0.0308	0.6503	0.1064	0.0176	0	0.0012	0.0164
**o27_31_bl**	o27	31	SC II	0.1911	0.0547	0.6552	0.0741	0.025	0	0.0012	0.0238
**o27_32_bl**	o27	32	SC II	0.2003	0.0434	0.6528	0.0778	0.0258	0	0.0008	0.025
**o27_33_bl**	o27	33	SC II	0.1755	0.0582	0.657	0.0751	0.0342	0	0.0012	0.033
**o27_34_bl**	o27	34	SC II	0.2464	0.0364	0.6217	0.0753	0.0201	0	0.001	0.0191
**o27_35_bl**	o27	35	SC II	0.266	0.0344	0.6092	0.0741	0.0163	0	0.0011	0.0152
**o27_36_bl**	o27	36	SC II	0.2091	0.0447	0.6501	0.0689	0.0273	0	0.0011	0.0262
**o27_37_bl**	o27	37	SC II	0.2088	0.039	0.6474	0.0789	0.0259	0	0.0014	0.0245

**o27_38_bl**	o27	38	SC II	0.2027	0.0475	0.6416	0.0787	0.0294	0	0.0012	0.0282
**o27_39_bl**	o27	39	SC II	0.1871	0.0486	0.6641	0.0755	0.0247	0	0.001	0.0237
**o27_40_bl**	o27	40	SC II	0.1915	0.038	0.6556	0.0966	0.0183	0	0.0012	0.0171
**o27_41_sl**	o27	41	SC II	0.2315	0.0425	0.6134	0.0786	0.034	0	0.0016	0.0324
**o27_42_sl**	o27	42	SC III	0.4355	0.0201	0.4841	0.0494	0.011	0	0.0021	0.0089
**o27_43_sl**	o27	43	SC II	0.2604	0.0406	0.5805	0.087	0.0314	0	0.001	0.0304
**o27_44_sl**	o27	44	SC II	0.2805	0.0358	0.5865	0.0746	0.0226	0	0.0014	0.0212
**o27_45_sl**	o27	45	SC II	0.2274	0.036	0.6148	0.0859	0.036	0	0.0016	0.0344
**o37_1_bl**	o37	46	SC III	0.3157	0.0347	0.5196	0.105	0.0251	0	0.0013	0.0238
**o37_2_bl**	o37	47	SC III	0.3538	0.0294	0.5195	0.0872	0.0102	0	0.0008	0.0094
**o37_3_bl**	o37	48	SC III	0.4024	0.0335	0.4829	0.0715	0.0097	0	0.001	0.0087
**o37_4_bl**	o37	49	SC III	0.3651	0.0258	0.5201	0.0752	0.014	0	0.0007	0.0133
**o37_5_bl**	o37	50	SC III	0.3752	0.0349	0.4673	0.0977	0.0249	0	0.0018	0.0231
**o37_6_bl**	o37	51	SC III	0.3834	0.0363	0.4776	0.0767	0.0261	0	0.001	0.0251
**o37_7_bl**	o37	52	SC III	0.2976	0.0424	0.5453	0.0808	0.0339	0	0.0014	0.0325
**o37_8_bl**	o37	53	SC III	0.3883	0.033	0.4701	0.0848	0.0239	0	0.0016	0.0223
**o37_9_bl**	o37	54	SC II	0.2174	0.0599	0.5387	0.0804	0.1036	0	0.0009	0.1027
**o37_10_bl**	o37	55	SC II	0.2296	0.0608	0.5586	0.0855	0.0654	0	0.0009	0.0645
**o37_11_bl**	o37	56	SC III	0.3641	0.0359	0.4759	0.0876	0.0364	0	0.0013	0.0351
**o37_12_bl**	o37	57	SC III	0.2882	0.0453	0.5435	0.0837	0.0394	0	0.0012	0.0382
**o37_13_bl**	o37	58	SC III	0.2958	0.0318	0.5939	0.0675	0.0109	0	0.0007	0.0102
**o37_14_bl**	o37	59	SC II	0.2641	0.0331	0.598	0.0849	0.0198	0	0.0008	0.019
**o37_15_bl**	o37	60	SC III	0.3464	0.0231	0.5304	0.091	0.0091	0	0.0012	0.0079
**o37_16_bl**	o37	61	SC III	0.3713	0.0205	0.4918	0.1021	0.0142	0	0.0019	0.0123
**o37_17_bl**	o37	62	SC III	0.3312	0.0421	0.5093	0.0854	0.032	0	0.0015	0.0305
**o37_18_bl**	o37	63	SC III	0.3015	0.0258	0.5803	0.0858	0.0067	0	0.0008	0.0059
**o37_19_bl**	o37	64	SC III	0.3141	0.0396	0.5235	0.0861	0.0366	0	0.0013	0.0353
**o37_20_bl**	o37	65	SC III	0.3785	0.033	0.4601	0.1012	0.0272	0	0.0012	0.026
**o37_21_bl**	o37	66	SC III	0.3826	0.0293	0.4706	0.0862	0.0313	0	0.0012	0.0301
**o37_22_bl**	o37	67	SC III	0.4107	0.0251	0.4615	0.0867	0.016	0	0.0011	0.0149
**o37_23_bl**	o37	68	SC III	0.334	0.0406	0.5105	0.0808	0.034	0	0.0013	0.0327
**o37_24_bl**	o37	69	SC III	0.3842	0.0246	0.4926	0.0858	0.0128	0	0.0005	0.0123
**o37_25_bl**	o37	70	SC III	0.3917	0.0262	0.4758	0.092	0.0143	0	0.0006	0.0137
**o38_1_bl**	o38	71	SC III	0.3326	0.0198	0.5287	0.1052	0.0138	0	0.0013	0.0125
**o38_2_bl**	o38	72	SC III	0.324	0.0234	0.5587	0.0791	0.0147	0	0.0017	0.013
**o38_3_bl**	o38	73	SC III	0.2485	0.0286	0.583	0.1273	0.0126	0	0.0016	0.011
**o38_4_bl**	o38	74	SC III	0.309	0.0271	0.5532	0.0946	0.0161	0	0.0014	0.0147
**o38_5_bl**	o38	75	SC III	0.3568	0.0236	0.5572	0.0487	0.0137	0	0.0017	0.012
**o38_6_bl**	o38	76	SC III	0.3117	0.0239	0.5461	0.0808	0.0375	0	0.0016	0.0359
**o38_7_bl**	o38	77	SC III	0.3562	0.0214	0.554	0.0552	0.0131	0	0.0012	0.0119
**o38_8_bl**	o38	78	SC III	0.3005	0.0291	0.5998	0.0563	0.0144	0	0.0016	0.0128
**o38_9_bl**	o38	79	SC III	0.3214	0.0221	0.5833	0.0594	0.0138	0	0.0016	0.0122
**o38_10_bl**	o38	80	SC III	0.2822	0.0329	0.6077	0.0616	0.0155	0	0.0015	0.014
**o38_11_bl**	o38	81	SC II	0.2598	0.0308	0.5897	0.0989	0.0209	0	0.0016	0.0193
**o38_12_bl**	o38	82	SC III	0.335	0.0284	0.5659	0.0578	0.0129	0	0.0017	0.0112
**o38_13_bl**	o38	83	SC III	0.3545	0.0214	0.5588	0.0513	0.0141	0	0.0019	0.0122
**o38_14_bl**	o38	84	SC III	0.3178	0.0279	0.5822	0.0579	0.0143	0	0.0016	0.0127

**o38_15_bl**	o38	85	SC III	0.3302	0.0309	0.5722	0.0472	0.0195	0	0.0055	0.014
**o38_16_bl**	o38	86	SC III	0.2666	0.025	0.588	0.1071	0.0134	0	0.0016	0.0118
**o38_17_bl**	o38	87	SC II	0.2549	0.0316	0.6015	0.0872	0.0247	0	0.0015	0.0232
**o38_18_bl**	o38	88	SC III	0.3099	0.0274	0.591	0.058	0.0137	0	0.0018	0.0119
**o38_19_bl**	o38	89	SC II	0.2674	0.0312	0.6192	0.0671	0.0151	0	0.0013	0.0138
**o38_20_bl**	o38	90	SC II	0.2409	0.0344	0.6162	0.0802	0.0283	0	0.001	0.0273
**o38_21_bl**	o38	91	SC III	0.2899	0.0346	0.594	0.0674	0.0141	0	0.001	0.0131
**o38_22_bl**	o38	92	SC II	0.2208	0.0417	0.573	0.1141	0.0504	0	0.0019	0.0485
**o38_23_bl**	o38	93	SC II	0.1967	0.0349	0.6199	0.1045	0.044	0	0.0017	0.0423
**o38_24_bl**	o38	94	SC III	0.3525	0.0264	0.5352	0.0731	0.0128	0	0.0011	0.0117
**o38_25_bl**	o38	95	SC II	0.2351	0.043	0.5769	0.0893	0.0557	0	0.0015	0.0542
**o38_26_bl**	o38	96	SC III	0.3924	0.02	0.4566	0.1156	0.0155	0	0.0016	0.0139
**o38_27_bl**	o38	97	SC II	0.2089	0.023	0.5871	0.1656	0.0154	0	0.0019	0.0135
**o38_28_bl**	o38	98	SC II	0.2417	0.032	0.6062	0.094	0.026	0	0.0018	0.0242
**o38_29_bl**	o38	99	SC III	0.2964	0.0349	0.5539	0.0862	0.0284	0	0.0089	0.0195
**o38_30_bl**	o38	100	SC III	0.3163	0.0206	0.5071	0.1429	0.013	0	0.0017	0.0113

Table S2. Supplementary. Pairwise comparisons for significant differences in the elemental composition of the spicule types.

**Linear regression model on the ILR scale (R^2 adj. = 0.7166891)**							
**Analysis of variance table**							
	Df	***Pillai***	***Approx. F***	***num Df***	***den Df***	***Pr (>F)***	
**(Intercept)**	1	0.99586	5657.8	4	94	$< 2.2e^{-16}$	***
**Clusters**	2	1.58641	91.1	8	190	$< 2.2e^{-16}$	***
**Residuals**	97						
**Contrast analysis**							
**Contrast**	**Estimate**	**SE**	**df**	***t*-ratio**	***p*-value**		
**C1–C2**	0.510	0.0370	97	13.770	<0.0001	***	
**C1–C2**	0.825	0.0366	97	22.519	<0.0001	***	
**C2–C3**	0.315	0.0258	97	12.177	<0.0001	***	

Table S3. Supplementary. Dataset of the elemental composition of the *Onchidoris muricata* spicules on fractures.

Original name of a spicule	Specimen	Index number of a spicule	Cluster	Ca	Mg	O	C	Traces	P	Cl	F
**o8_1_un**	o8	101	FC II	0.2426	0.0361	0.61	0.0943	0.0169	0.0000	0.0011	0.0158
**o8_2_un**	o8	102	FC III	0.1776	0.0578	0.5807	0.1248	0.059	0.0000	0.0008	0.0582
**o8_3_un**	o8	103	FC II	0.2795	0.0365	0.4709	0.1667	0.0465	0.0000	0.0015	0.045
**o8_4_un**	o8	104	FC III	0.1238	0.0873	0.6208	0.0872	0.0809	0.0000	0.0007	0.0802
**o8_5_un**	o8	105	FC I	0.3641	0.0309	0.3997	0.0885	0.1169	0.1079	0.0018	0.0072
**o8_6_un**	o8	106	FC II	0.2214	0.0238	0.5587	0.1708	0.0252	0.0000	0.0016	0.0236
**o8_7_un**	o8	107	FC III	0.1302	0.0624	0.6285	0.1259	0.053	0.0000	0.0007	0.0523
**o8_8_un**	o8	108	FC III	0.2061	0.0502	0.545	0.1167	0.082	0.0548	0.0008	0.0264
**o8_9_un**	o8	109	FC IV	0.2304	0.0259	0.4153	0.1875	0.1409	0.1308	0.0008	0.0093
**o8_10_un**	o8	110	FC III	0.2142	0.0609	0.6019	0.0705	0.0526	0.0000	0.0009	0.0517
**o8_11_un**	o8	111	FC II	0.2785	0.0431	0.5014	0.1194	0.0575	0.0435	0.0006	0.0134
**o8_12_un**	o8	112	FC II	0.24	0.0397	0.5648	0.1148	0.0408	0.0000	0.0009	0.0399
**o8_13_un**	o8	113	FC II	0.283	0.0503	0.4638	0.1846	0.0182	0.0000	0.0012	0.017
**o8_14_un**	o8	114	FC II	0.2622	0.0526	0.5767	0.0935	0.0149	0.0000	0.0013	0.0136
**o8_15_un**	o8	115	FC II	0.1825	0.0293	0.5931	0.1773	0.0177	0.0000	0.0007	0.017
**o8_16_un**	o8	116	FC II	0.3335	0.0125	0.537	0.1021	0.0149	0.0044	0.0000	0.0105
**o8_17_un**	o8	117	FC II	0.2753	0.0326	0.5701	0.0943	0.0277	0.0000	0.0015	0.0262
**o8_18_un**	o8	118	FC II	0.4599	0.0202	0.4599	0.051	0.0088	0.0000	0.0015	0.0073
**o8_19_un**	o8	119	FC III	0.1106	0.0533	0.7037	0.1063	0.0261	0.0000	0.0003	0.0258
**o8_20_un**	o8	120	FC III	0.1914	0.066	0.558	0.1051	0.0794	0.0000	0.0011	0.0783
**o8_21_un**	o8	121	FC III	0.1038	0.0639	0.6307	0.1219	0.0797	0.0000	0.0004	0.0793
**o8_22_un**	o8	122	FC II	0.343	0.0367	0.49	0.1076	0.0228	0.0000	0.0014	0.0214
**o8_23_un**	o8	123	FC II	0.3071	0.0502	0.5539	0.0678	0.021	0.0000	0.0012	0.0198
**o8_24_un**	o8	124	FC II	0.3071	0.0258	0.5539	0.0982	0.015	0.0000	0.0015	0.0135
**o8_25_un**	o8	125	FC II	0.2176	0.0234	0.6093	0.1305	0.0192	0.0000	0.0012	0.018
**o4_1_un**	o4	126	FC III	0.1991	0.0379	0.6149	0.1044	0.0437	0.0000	0.0009	0.0428
**o4_2_un**	o4	127	FC III	0.1793	0.0554	0.628	0.1014	0.0358	0.0000	0.0008	0.035
**o4_3_un**	o4	128	FC III	0.1474	0.0525	0.6678	0.1029	0.0295	0.0000	0.0004	0.0291
**o4_4_un**	o4	129	FC II	0.2276	0.0372	0.6393	0.0815	0.0144	0.0000	0.0007	0.0137
**o4_5_un**	o4	130	FC IV	0.211	0.0387	0.4448	0.1543	0.1512	0.1394	0.0008	0.011
**o4_6_un**	o4	131	FC II	0.5033	0.0178	0.3412	0.1314	0.0063	0.0000	0.001	0.0053
**o4_7_un**	o4	132	FC I	0.3415	0.0132	0.4254	0.099	0.1209	0.1131	0.0011	0.0067
**o4_8_un**	o4	133	FC II	0.1836	0.0477	0.6395	0.1114	0.0178	0.0000	0.0003	0.0175
**o4_9_un**	o4	134	FC II	0.2336	0.0119	0.5378	0.2064	0.0103	0.0000	0.0008	0.0095
**o4_10_un**	o4	135	FC III	0.2042	0.0502	0.6264	0.0943	0.025	0.0000	0.0007	0.0243
**o4_11_un**	o4	136	FC II	0.2733	0.0335	0.5692	0.1	0.0239	0.0000	0.0012	0.0227
**o4_12_un**	o4	137	FC III	0.2268	0.0231	0.5647	0.0997	0.0857	0.0733	0.0006	0.0118
**o4_13_un**	o4	138	FC I	0.3029	0.0317	0.3107	0.147	0.2078	0.2027	0.0011	0.004
**o4_14_un**	o4	139	FC III	0.1947	0.0519	0.6366	0.0861	0.0307	0.0000	0.0007	0.03
**o4_15_un**	o4	140	FC II	0.45	0.0376	0.4374	0.0508	0.0242	0.0000	0.0009	0.0233

**o4_16_un**	o4	141	FC II	0.2965	0.048	0.5381	0.0736	0.0438	0.0000	0.0004	0.0434
**o4_17_un**	o4	142	FC II	0.2656	0.0567	0.5734	0.0719	0.0322	0.0000	0.0008	0.0314
**o4_18_un**	o4	143	FC III	0.1674	0.0724	0.6441	0.0772	0.039	0.0000	0.0006	0.0384
**o4_19_un**	o4	144	FC IV	0.0845	0.0443	0.649	0.1487	0.0735	0.0312	0.0004	0.0419
**o4_20_un**	o4	145	FC III	0.1483	0.0421	0.6614	0.1195	0.0286	0.0088	0.0001	0.0197
**o4_21_un**	o4	146	FC II	0.2707	0.0785	0.4801	0.134	0.0367	0.0000	0.0013	0.0354
**o4_22_un**	o4	147	FC III	0.1498	0.0467	0.6489	0.1116	0.043	0.026	0.0002	0.0168
**o4_23_un**	o4	148	FC II	0.4025	0.0231	0.3981	0.1692	0.0071	0.0000	0.0014	0.0057
**o4_24_un**	o4	149	FC III	0.1272	0.0669	0.5759	0.136	0.094	0.0000	0.0005	0.0935
**o4_25_un**	o4	150	FC II	0.2692	0.03	0.4279	0.2253	0.0476	0.0000	0.0014	0.0462
**o38_1_un**	o38	151	FC I	0.3862	0.0037	0.2347	0.2376	0.1378	0.1314	0.0043	0.0021
**o38_2_un**	o38	152	FC IV	0.2431	0.0254	0.4251	0.1354	0.1711	0.1572	0.0079	0.006
**o38_3_un**	o38	153	FC IV	0.2917	0.0057	0.4646	0.1771	0.061	0.05	0.0048	0.0062
**o38_4_un**	o38	154	FC I	0.3198	0.0061	0.3256	0.2199	0.1285	0.1229	0.0016	0.004
**o38_5_un**	o38	155	FC IV	0.249	0.0245	0.394	0.1438	0.1886	0.1797	0.0015	0.0074
**o38_6_un**	o38	156	FC IV	0.1705	0.0253	0.5172	0.144	0.143	0.1286	0.002	0.0124
**o38_7_un**	o38	157	FC I	0.3049	0.0093	0.3112	0.2242	0.1503	0.1432	0.0027	0.0044
**o38_8_un**	o38	158	FC I	0.4236	0.0079	0.1948	0.1739	0.1999	0.1956	0.002	0.0023
**o38_9_un**	o38	159	FC IV	0.3101	0.0076	0.4386	0.1463	0.0974	0.0893	0.0013	0.0068
**o38_10_un**	o38	160	FC IV	0.0906	0.0277	0.5925	0.1844	0.1048	0.0852	0.001	0.0186
**o38_11_un**	o38	161	FC I	0.3184	0.0295	0.2931	0.1393	0.2198	0.2108	0.0027	0.0063
**o38_12_un**	o38	162	FC II	0.3974	0.007	0.4608	0.1269	0.0079	0.0000	0.0008	0.0071
**o38_13_un**	o38	163	FC I	0.4016	0.0034	0.2815	0.1744	0.1392	0.1355	0.0013	0.0024
**o38_14_un**	o38	164	FC I	0.2889	0.0157	0.32	0.1956	0.1797	0.1726	0.0018	0.0053
**o38_15_un**	o38	165	FC IV	0.1057	0.0285	0.5916	0.1605	0.1138	0.0958	0.001	0.017
**o38_16_un**	o38	166	FC IV	0.1919	0.0226	0.4452	0.1919	0.1485	0.1361	0.0021	0.0103
**o38_17_un**	o38	167	FC IV	0.1842	0.025	0.4715	0.1682	0.1511	0.1385	0.0016	0.011
**o38_18_un**	o38	168	FC IV	0.1034	0.0301	0.5841	0.1709	0.1116	0.0945	0.0011	0.016
**o38_19_un**	o38	169	FC IV	0.2004	0.0194	0.4727	0.1701	0.1372	0.1258	0.0018	0.0096
**o38_20_un**	o38	170	FC IV	0.1313	0.0218	0.5639	0.1791	0.1039	0.0938	0.0013	0.0088
**o38_21_un**	o38	171	FC IV	0.2719	0.0213	0.3971	0.1353	0.1743	0.1674	0.0014	0.0055
**o38_22_un**	o38	172	FC IV	0.1705	0.0204	0.517	0.1581	0.1338	0.1224	0.0015	0.0099
**o38_23_un**	o38	173	FC IV	0.1239	0.0272	0.5455	0.1817	0.1218	0.1051	0.0014	0.0153
**o38_24_un**	o38	174	FC IV	0.0895	0.0252	0.5914	0.1897	0.104	0.0855	0.0011	0.0174
**o38_25_un**	o38	175	FC IV	0.136	0.0264	0.5182	0.1859	0.1335	0.1164	0.0015	0.0156
**o38_26_un**	o38	176	FC IV	0.1594	0.0237	0.5027	0.1693	0.145	0.1318	0.0016	0.0116
**o38_27_un**	o38	177	FC I	0.272	0.028	0.3441	0.1639	0.1921	0.1869	0.0016	0.0036
**o38_28_un**	o38	178	FC IV	0.1091	0.0267	0.5769	0.1718	0.1155	0.1028	0.0008	0.0119
**o38_29_un**	o38	179	FC III	0.2222	0.0351	0.5877	0.0952	0.0598	0.0458	0.0009	0.0131
**o38_30_un**	o38	180	FC IV	0.1005	0.0253	0.5787	0.1877	0.1077	0.0917	0.0014	0.0146
**o48_1_un**	o48	181	FC IV	0.1119	0.0223	0.6074	0.1358	0.1226	0.1	0.0009	0.0217
**o48_2_un**	o48	182	FC IV	0.1268	0.0229	0.5782	0.1563	0.1158	0.1011	0.0015	0.0132
**o48_3_un**	o48	183	FC IV	0.1763	0.0234	0.5252	0.1244	0.1508	0.139	0.0008	0.011
**o48_4_un**	o48	184	FC IV	0.0991	0.0225	0.6224	0.1537	0.1023	0.087	0.0008	0.0145
**o48_5_un**	o48	185	FC I	0.344	0.0086	0.3706	0.1079	0.1688	0.1642	0.0013	0.0033
**o48_6_un**	o48	186	FC IV	0.127	0.0243	0.578	0.152	0.1187	0.1051	0.0016	0.012
**o48_7_un**	o48	187	FC IV	0.1637	0.019	0.5248	0.1632	0.1292	0.1146	0.0015	0.0131

**o48_8_un**	o48	188	FC IV	0.129	0.0151	0.5935	0.1944	0.068	0.0412	0.0018	0.025
**o48_9_un**	o48	189	FC IV	0.1472	0.025	0.5545	0.1391	0.1342	0.1218	0.001	0.0114
**o48_10_un**	o48	190	FC IV	0.1021	0.0197	0.5765	0.2058	0.0959	0.0772	0.0014	0.0173
**o48_11_un**	o48	191	FC IV	0.2232	0.0196	0.4473	0.1473	0.1625	0.1514	0.0013	0.0098
**o48_12_un**	o48	192	FC I	0.356	0.0129	0.2425	0.1919	0.1967	0.1912	0.0017	0.0038
**o48_13_un**	o48	193	FC IV	0.315	0.0012	0.4287	0.2149	0.0403	0.0395	0.0008	0
**o48_14_un**	o48	194	FC I	0.4732	0.005	0.2635	0.0832	0.175	0.1698	0.0014	0.0038
**o48_15_un**	o48	195	FC I	0.2816	0.0332	0.3358	0.1162	0.2331	0.2262	0.002	0.0049
**o48_16_un**	o48	196	FC IV	0.2458	0.0129	0.4354	0.2078	0.0981	0.0889	0.0012	0.008
**o48_17_un**	o48	197	FC IV	0.2388	0.0301	0.3875	0.1301	0.2136	0.206	0.002	0.0056
**o48_18_un**	o48	198	FC IV	0.0948	0.0209	0.579	0.2108	0.0944	0.0803	0.0013	0.0128
**o48_19_un**	o48	199	FC IV	0.1271	0.0247	0.5679	0.183	0.0972	0.0793	0.0027	0.0152
**o48_20_un**	o48	200	FC IV	0.1248	0.0264	0.5783	0.1463	0.1242	0.1102	0.0015	0.0125
**o48_21_un**	o48	201	FC IV	0.1195	0.0002	0.5709	0.2952	0.0141	0	0.0012	0.0129
**o48_22_un**	o48	202	FC I	0.5141	0.0094	0.1249	0.1113	0.2403	0.2367	0.0029	0.0007
**o48_23_un**	o48	203	FC I	0.2692	0.026	0.3497	0.1681	0.1869	0.1798	0.0019	0.0052
**o48_24_un**	o48	204	FC I	0.3437	0.0288	0.2659	0.1355	0.2261	0.2194	0.0022	0.0045
**o48_25_un**	o48	205	FC I	0.5465	0.0034	0.2429	0.0856	0.1216	0.1189	0.0005	0.0022
