# Supplementary figures and images for: Mineral Composition of Skeletal Elements in Dorid Nudibranchia Onchidoris muricata (Gastropoda, Mollusca)
Source: Biomimetics (Basel). 2025 Mar 29;10(4):211. doi: 10.3390/biomimetics10040211 (PMC12025115; doi:10.3390/biomimetics10040211)

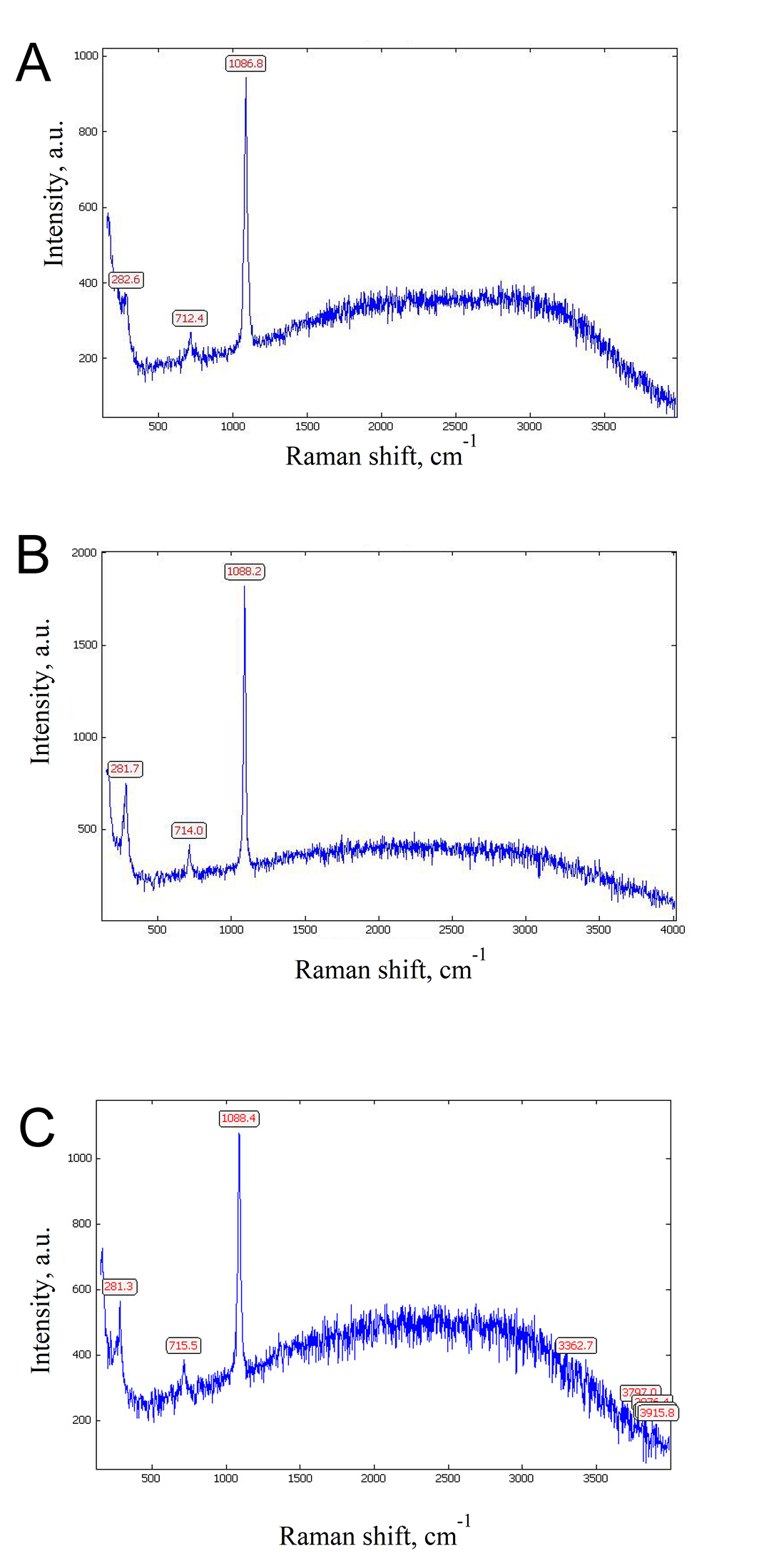

Supplement: Supplementary file 1 [file biomimetics-10-00211-s001.zip › Figure S1.tif]
